# Foreign Correspondence

**Published:** 1838-10-10

**Authors:** 


### FOREIGN CORRESPONDENCE.

*Observations on Chronic Peritonitis and Double Pleurisy.* By P. Ch. A. Louis, M. D., &c., &c., *Physician to the Hospital of La Pitié, at Paris.*

Chronic peritonitis is an affection of great frequency; in thirty-nine cases of phthisis, I found it present in seven, and in a much greater proportion of women than of men. For, of the seven cases in question, four were of women out of nine phthisical patients, and the other three were of men out of thirty. You know that I refer, in this analysis, to facts collected at La Pitié, in the course of my clinical researches.

Chronic peritonitis I have noticed only in tuberculous subjects, and always as the result of the developement of tubercles, either beneath or upon the surface of the peritoneum; chronic peritonitis and tubercular peritonitis, I therefore look upon as synonymous terms. Again, from the law, that tubercles cannot, after the age of fifteen, exist in any organ, without being also in the lungs, we must, in all well established cases of chronic peritonitis, conclude the co-existence of pulmonary tubercles. This is an important law, which serves to prevent a disease, beyond the resources of art, from being confounded with one of a trifling character; and is, hence, an eminently practical law, as it is one of frequent application. The following fact is, I think, the first of the kind, which offered itself to your observation, and that of our lamented friend, Jackson, and in which I recognised the tuberculous affection from the presence of chronic peritonitis.

A labourer, eighteen years of age, of a tolerably strong constitution, was brought to the hospital of La Pitié, ward St. Paul, the 22d of March, 1831, having been at that time ill five months and a half. His first symptoms had been a well-marked febrile movement, with cephalalgia, cough, and diarrhœa. He almost immediately entered the hospital of La Charité, where he remained five months. His cough, while he was there, lasted for a month, and afterwards disappeared. So also with his diarrhœa: for the first four weeks, the patient had four or five liquid stools a day; but he had afterwards only two during the same time, and of tolerable consistency. Anorexia was nearly complete during the first four months; the fifth, the patient ate a half or a quarter of his daily allowance. His strength remained throughout in a state of extreme prostration. He never had hemoptysis, or pains in the breast or belly.

His condition on the 23d of March, the day after his admission into La Pitié, was the following:—Intelligence ordinary; universal pallor; skin dry and rugous; heat of the body slightly elevated, not felt by the patient; abdomen tympanitic below the umbilicus, equally elastic in this part; as free from pain or pressure as formerly; one stool the night before; appetite almost gone; took bread and some soup; respiration moderately accelerated, without cough. Percussion obscure, from the right clavicle to the nipple, and still more so below. Respiration feeble in the same spot, without gurgling or any sort of rhonchus. Posteriorly, auscultation and percussion furnished the same results. On the left side, nothing remarkable. Pulse one hundred and four. Considerable emaciation.

The patient died twelve days after his admission into the hospital, having had cough only for the last five, and after the developement of cerebral symptoms, which appeared eight days before dissolution. These symptoms were principally epileptic paroxysms, which were renewed several times during the first four days of the period in question. During the same interval, the intelligence was obtuse; debility more marked than before their developement; the expression of the features altered; the indifference of the patient to surrounding objects was extreme. The symptoms persisted, with some variations, until death. A slight sub-crepitant rhonchus, under the right clavicle, was noticed on the night preceding death.

At the autopsy, thirty hours after death, the appearances were the following:—*Brain*, on the right side, at the junction of the posterior and middle lobes, red softening for the space of a cubic inch, surrounded by a white softening, comprising the cineritious substance; some small tubercles in the neighbourhood, confined to the cineritious substance. The pia mater every where infiltrated with a puriform or tuberculous matter. A similar softening, of not quite the same extent, on the left side, at the level of the optic thalamus. Septum lucidum semi-transparent, extremely softened, and loose at its inferior extremity. Two spoonfuls of serosity in each of the lateral ventricles. Nothing else remarkable. *Neck*—some cervical or bronchial glands tuberculous. Pharynx, larynx, and trachea, normal. *Heart* slightly softened, otherwise healthy, surrounded by two ounces of serosity in the pericardium; aorta slightly tinged with a reddish hue. *Thorax*—eight ounces of pure serum in the left pleura, which was free. Universal adhesions on the right side, with a very thick tuberculous false membrane in the inferior half. Numerous small tubercles, some slightly softened, in the two superior lobes. Bronchi healthy. *Abdomen*—strong adhesion of the omentum majus to the anterior parietes of the abdomen, and of the liver and spleen to the surrounding parts, through the medium of tubercles, the size of a pea, or a little larger. Numerous tubercles over the surface of the intestines, of the bladder, and of the mesentery, which were agglutinated together. Two perforations of the stomach, one on its great cul-de-sac, and the other anteriorly, one inch from the great curvature; the latter eight or ten inches broad; the other much more considerable. Interiorly, contraction of the mucous membrane around the perforation of the great cul-de-sac; and anteriorly, in a corresponding spot; and in another, where only the serous membrane remained, for a surface of four inches. Elsewhere, the gastric mucous membrane presented its ordinary velvety appearance, and, in three-fourths or two-thirds of its extent, had a normal consistence. The kidneys and liver were natural, and the spleen, with the exception of some tubercles. The stomach contained but a small quantity of a yellowish, mucous liquid, and the surrounding parts offered no traces of recent inflammations. The mucous membrane of the small intestine was healthy, except a slight softening in the lowest foot; that of the large intestine was slightly softened and thickened, in the two first feet, elsewhere normal. The bladder offered nothing remarkable.

Evidently, in this case, the signs of auscultation and percussion were insufficient to point out the alteration in the parenchyma of the lungs. From the extent of the space in which the sonorousness of the right side was altered, without the complication of any rhonchus, (except in the last two days of life,) we had even reason to attribute the obscurity of the sound to the presence of a thick false membrane, which was in part the case. Notwithstanding the absence of cough and diarrhœa, the emaciation served to put us in the way of a diagnosis, although not to establish the certainty of the existence of a tubercular affection. But, the condition of the abdomen, which was voluminous, resisting, uniformly elastic and sonorous, as in chronic peritonitis, a condition which could be traced to the commencement of the illness, could be considered only as the result of chronic peritonitis, (although the patient complained of no pain in the belly,) and could be ascribed only to a tuberculous disease. In this case, as I pointed out before, percussion and auscultation, generally so useful, served rather as sources of error, than the means of establishing the truth. To arrive at a correct diagnosis, it was necessary to take into consideration the progress of the disease, the emaciation, the state of the abdomen, &c. &c.

*Double Pleurisy.*—This affection I have observed only in individuals whose lungs were more or less considerably diseased, from some organic affection, the cure of which was hopeless; and, with a single exception, all the subjects of my observation were tuberculous. The exception was the case of a young subject, who, shortly after a trifling operation performed upon him by M. Velpeau, while apparently in a state of perfect health, was suddenly seized with pain on the right, and, afterwards, on the left side of the chest. The existence of a double effusion was soon ascertained, and the prognosis was consequently unfavourable. After an illness of eight days, the patient died; and an examination of the body disclosed a partial and circumscribed gangrene of both lungs, beneath the pleuræ.*

With regard to the prognosis of tubercular affections, the necessity of carefully watching the course of the disease, in forming an estimate of its probable termination, is all-important. If it has advanced slowly—if, after a duration of two or three years, it is accompanied with inconsiderable constitutional derangement, and with little or no fever, it may be indefinitely prolonged, before ending in death. Under the same point of view of prognosis, it is of consequence to determine whether the patient has had a pleurisy on both sides of the chest, since, if he have, no danger is to be apprehended of a perforation of the pleura, one of the most formidable accidents which complicates tubercular affections, and which, unfortunately, is not of uncommon occurrence.

*Hemoptysis.*—In a period of ten years, I have seen but a solitary case of hemoptysis at all severe, in an individual not tuberculous. This case was of a young woman, who died of a typhoid affection, after having spit up, in the space of about an hour, from three to four ounces of blood. The lungs were perfectly healthy.

*Fatty Liver.*—During the same period, I have met with but a single fatty liver, in an individual not tuberculous; and all the facts which I have collected, confirm the disproportion, existing in this respect, between the sexes, which I have laid down in my work on phthisis. I may say the same of *ulcerations* of the pharynx, œsophagus, epiglottis, larynx, trachea, bronchi, and of the small intestine: these ulcerations occur, during the course of a chronic disease, only in tuberculous patients. It is worthy of remark, that ulcerations of the bronchi do not take place, even in prolonged inflammation of these organs, when they are dilated, thickened, and of a deep red colour, &c. &c., unless there are tubercles in the lungs.

*Perforation* of the small intestine I have met with in phthisical patients, only twice, in ten years: it is, therefore, an accident of very rare occurrence.

I have met with some cases of chronic peritonitis, still more interesting than those whose history I have previously given you, inasmuch as one of the patients, to whom I refer, came to the hospital eight or ten days from the commencement of the disease, having at the time only the symptoms of chronic peritonitis, without any alteration in the sonorousness of the chest, or in the character of the respiratory sound. Having been relieved from her sufferings, she left the hospital after a stay of about two weeks, and returned, at the end of two months, with pains in the abdomen, which was voluminous and resisting. She then offered a trifling alteration in the respiratory sound, under the right clavicle, with slight bronchophony and bronchial respiration, posteriorly, on the same side. This woman returned three times to the hospital, for the same affection, in the course of two years, not much emaciated, with little or no fever. I know not what finally became of her, but she was manifestly tuberculous, and it was the chronic peritonitis which enabled me from the first to recognise the affection.

*Treatment.*—I should insist particularly upon the importance of opiates, to allay the irritation of the cough; how much relief is experienced by many patients, treated without opium by their physician, when it is prescribed for them! I have confidence in fumigations with narcotic or mucilaginous plants. Many patients, without appetite, and suffering from nausea, derive marked advantage from the artificial Seltzer water, which I have found to excite coughing, in no one instance in which I have administered it. I am acquainted with no fact, which proves the utility of blisters on the arms, or of issues in the same spot, or below the clavicles. A short time since, at a consultation of six physicians, at which Drs. Fouquier and Chomel were present, not one was able to cite any facts in support of the good effects of drains in arresting the course of a tubercular affection. As regards chlorine, it has often had the credit of the cure of a phthisis, which was only a pulmonary catarrh. That such a mistake may take place has been proved by a recent case, in which death having accidentally supervened, no lesion of the lung was found, but a partial dilatation of the bronchi, at the summit of one of the lungs, which, during life, was the cause of the phenomenon of bronchophony.

*Seat of the Disease and Diagnosis.*—It is to be noted, that, when pulmonary catarrh gives rise to

*This pathological relation between double pleurisy and disorders of the lung, may be thus stated. Double pleurisy may be regarded as always consecutive to an organic disease of the lung, which in the immense majority of cases of chronic disease, is tubercular. Still, all other affections of the parenchyma give rise to the same complication.—*Eds.*

the sub-crepitant rhonchus, which is the most common, this rhonchus is seated posteriorly and inferiorly in the chest; whilst, in phthisis, when the respiration begins to undergo alteration, it is at the summit of the chest that this alteration commences. I know no exception to this law, which I have established from at least one hundred and fifty cases, in which the pulmonary catarrh was simple, or complicated, by being developed during the course of an eruptive disease, or of a typhoid affection, or of an emphysema of the lungs. Phthisis and pulmonary catarrh do not, it may be laid down as a law, occupy the same situations in the chest.

[These remarks of Dr. Louis upon some interesting points, connected with the subject of tubercular affections, are intended as supplementary to his work on phthisis, with which most of our readers must be familiar. The general experience of the profession during the period that has elapsed since the publication of this work, (1825,) has confirmed all the essential facts then announced by Dr. Louis, and fully borne him out in the important laws which he laid down respecting the pathology of tubercular phthisis. The additional illustrations, which his own subsequent experience has collected, and which are now for the first time published, bear, it will be seen, upon some of the more intricate questions involved in the subject, and, although somewhat desultory in the mode in which they are arranged, and partaking of the familiarity of the epistolary style, are evidently the result of deep reflection and the most cautious analysis.—Eds.]

